# Evaluation of Clinical Meaningfulness of Red Clover (*Trifolium pratense* L.) Extract to Relieve Hot Flushes and Menopausal Symptoms in Peri- and Post-Menopausal Women: A Systematic Review and Meta-Analysis of Randomized Controlled Trials

**DOI:** 10.3390/nu13041258

**Published:** 2021-04-11

**Authors:** Wiesław Kanadys, Agnieszka Barańska, Agata Błaszczuk, Małgorzata Polz-Dacewicz, Bartłomiej Drop, Krzysztof Kanecki, Maria Malm

**Affiliations:** 1 Department of Informatics and Medical Statistics, Medical University of Lublin, 20-090 Lublin, Poland; wieslaw.kanadys@wp.pl (W.K.); bartlomiej.drop@umlub.pl (B.D.); maria.malm@umlub.pl (M.M.); 2 Department of Virology with SARS Laboratory, Medical University of Lublin, 20-093 Lublin, Poland; agata.blaszczuk@umlub.pl (A.B.); malgorzata.polz.dacewicz@umlub.pl (M.P.-D.); 3 Department of Social Medicine and Public Health, Warsaw Medical University, 02-007 Warsaw, Poland; kkanecki@wum.edu.pl

**Keywords:** red clover, isoflavones, Trifolium pratense, hot flushes, menopausal symptoms, postmenopausal women, perimenopausal women

## Abstract

The meta-analysis presented in this article covered the efficacy of red clover isoflavones in relieving hot flushes and menopausal symptoms in perimenopausal and postmenopausal women. Studies were identified by MEDLINE (PubMed), Embase, and the Cochrane Library searches. The quality of the studies was evaluated according to Cochrane criteria. A meta-analysis of eight trials (ten comparisons) demonstrated a statistically significant reduction in the daily incidence of hot flushes in women receiving red clover compared to those receiving placebo: weighted mean difference (WMD—weighted mean difference) −1.73 hot flushes per day, 95% CI (confidence interval) −3.28 to −0.18; *p* = 0.0292. Due to 87.34% homogeneity, the performed analysis showed substantive difference in comparisons of postmenopausal women with ≥5 hot flushes per day, when the follow-up period was 12 weeks, with an isoflavone dose of ≥80 mg/day, and when the formulations contained a higher proportion of biochanin A. The meta-analysis of included studies assessing the effect of red clover isoflavone extract on menopausal symptoms showed a statistically moderate relationship with the reduction in the daily frequency of hot flushes. However, further well-designed studies are required to confirm the present findings and to finally determine the effects of red clover on the relief of flushing episodes.

## 1. Introduction

Menopause is characterized by amenorrhea due to the cessation of ovarian function. The decrease in circulating estrogens levels can induce menopausal disorders, including shorter-term symptoms, such as vasomotor symptoms, palpitations, sleep difficulties, headaches, fatigue, mood disturbances, and impaired concentration, and longer-term chronic conditions, such as cardiovascular diseases, accelerated bone loss, and cognitive impairment [1,2].

Hot flushes (HFs) are the most common symptom of menopause in about 70% of women, with differences in different populations, and may persist for several years after menopause [3,4]. The frequency of these symptoms depends on several factors, including climate, race/ethnicity, diet, lifestyle, women’s roles, and attitudes regarding the end of reproductive life and aging [5,6,7]. They can affect not only the quality of life, but also contribute to sleep and mood disturbance, which can potentially affect daily activities at home and at work to such an extent that treatment is required [8,9]. HFs are thought to be the result of the brain’s response to a progressive estrogen deficiency and fluctuation in the activity of neurotransmitters, especially in the serotonergic and noradrenergic pathways, which leads to instability of the mechanism of thermoregulation in the hypothalamus. Ultimately, this leads to increased blood flow through the skin and increased sweat gland activity, and as a result, these symptoms appear [10,11].

Despite the well-known benefits of menopausal hormonal therapy (HT), due to potentially serious side effects and breast cancer risk, the use of this therapy, even in the treatment of HFs, remains controversial [12,13]. Many women discontinued HT after the publication of the results of the Women's Health Initiative, looking for an effective and safe alternative to relieve menopausal symptoms [14,15]. The lack of acceptance of HT, related to concerns about its safety, has led to the popularization of many alternative and complementary methods of treatment [16,17,18]. For some years, red clover has been one such alternative used by women to treat vasomotor symptoms of menopause [19].

Red clover (*Trifolium pratense L., Fabaceae*) mainly contains the isoflavone aglycones, formononetin, and biochanin A; other isoflavones, such as genistein, daidzein, glycitein, and prunetin, were also identified in small quantities [19,20]. The mentioned methoxy precursors in the intestine and liver are demethylated by cytochrome P450 isozymes to the active forms, genistein and daidzein [21]. Red clover isoflavones with structural similarities to the endogenous 17β-estradiol reveal their biological effects via activating estrogen receptors (ER), with a higher affinity to ER-β in comparison to ER-α. In addition, a number of non-hormonal effects have been reported in isoflavones, including tyrosine kinase inhibition, antioxidant activity, and effects on ion transport [18,22,23]. In addition, a number of non-hormonal effects have been reported in isoflavones, including antioxidant activity, tyrosine kinase inhibition, and effects on ion transport [18,22,23].

This systematic review with meta-analysis aimed to clarify whether supplementation of red clover isoflavone extract (RCIE) affects menopausal symptoms in perimenopausal and postmenopausal women.

## 2. Materials and Methods

### 2.1. Search Strategy and Study Selection

To determine if intervention with the RCIE, as compared to placebo, relieves HFs, we reviewed published clinical trials in accordance with the PRISMA (The Preferred Reporting Items for Systematic Reviews and Meta-Analyses) guidelines [24]. The electronic databases MEDLINE (PubMed), Embase, and the Cochrane Library were searched for the identification of randomized controlled trials from 1999 to January 2020. The following search terms were used for all databases in various combination: “menopause” or “perimenopause” or “postmenopause” AND “red clover” or “Trifolium pratense” or “isoflavone” AND “hot flushes” or “menopausal symptoms”. The search was limited to the articles published in the English language for full analysis. References to selected research and review articles related to the topic of the work were also searched in order to identify additional studies.

### 2.2. Inclusion and Exclusion Criteria

Studies were considered eligible for inclusion if they met all of the following criteria: (a) parallel-group controlled trials; (b) trials with a crossover design that contained data for the first period; (c) comparison with placebo; (d) perimenopausal and postmenopausal women; (e) experiencing moderate to severe HFs at least three times per day in a two-week follow-up before the study entry; (f) primary outcomes that were changes in frequency of HFs per day, obtained by self-report using symptom diaries; (g) secondary outcomes that were the cumulative rating of menopausal symptoms using a questionnaire based on the respondents’ replies concerning the intensity of complaints. The used questionnaires and their descriptions are as follows: the Kupperman Menopausal Index (KMI) is a measure using a list of 11 symptoms (hot flushes, excessive sweating, sleep disturbances, irritability, depressive mood, attention deficit disorder, joint and bone pain, headache, arrhythmias, paresthesia) rated on a 4-point severity scale [25]. The Greene Climacteric Scale (GCS) is a tool based on a list of 21 symptoms rated on a 4-point Likert scale. The symptoms are divided into three categories: (a) psychological—anxiety (heart beating fast or strong, feeling tense or nervous, difficulty sleeping, being agitated, having anxiety or panic attacks, difficulty concentrating) and depression (feeling tired or lacking energy, loss of interest in most things, feeling unhappy or depressed, crying, irritable); (b) somatic (dizziness or fainting, pressure or tightness in the head, numbness in part of the body, headaches, aches and pains in the muscles and joints, loss of feeling in the hands or feet, difficulty breathing); (c) vasomotor (hot flushes, sweating at night); with an additional question related to sexual dysfunction (loss of interest in sex) [26]. The Menopause Rating Scale (MRS) consists of 11 items divided into three subscales, i.e., sweating/hot flushes, heart discomfort, sleep problems, joint and muscle problems, classified as somatic-vegetative symptoms; depressed mood, irritability, restlessness, and physical/mental exhaustion, classified as psychological symptoms; and sexual problems, bladder problems, and vaginal dryness, classified as urogenital symptoms [27].

Studies were excluded if they were duplicated reports, the duration of the study was less than 12 weeks, RCIE was combined with other plant medicines, lacked sufficient information, and if results were presented as graphics or percentage changes.

### 2.3. Data Extraction

The data were collected by the main author and then checked by the co-authors for correctness. The extracted data included the name of the first author; year of publication; country of origin; study design; follow-up period of the study; number of participants (randomized/analyzed); age (range) of women; daily dose of RCIE in the active arm (aglycone equivalent); a clearly described isoflavone component and their daily doses; baseline and final frequencies of HFs per day; scores of menopausal symptoms (without distinction between types of symptoms) or their differences; standard deviation, standard error, or 95% confidence intervals; and group size in each test arm.

### 2.4. Quality Assessment and Bias Risk of the Trials

The Cochrane risk of bias tool consists of seven items, which have a potential biasing influence on the estimates of an interventions’ effectiveness in randomized trials: selection bias, performance bias, detection bias, attrition bias, reporting bias, and a catch-all item called “other sources of bias”. The risk of bias in RCTs (randomized controlled trials) are included in the review as “High risk”, “Unclear”, or “Low risk” [28].

### 2.5. Statistical Analysis

The meta-analysis included all intervention groups from multi-arm studies. Moreover, to avoid duplication of data from the same people in surveys covering multiple time points, only one of such points was taken into account. The outcome measures were the difference in mean (MD) frequency of HFs between baseline and the end of the treatment, for both the intervention and control groups. Data of the size of the effects of RCIE in each study were presented as number of subjects (*n*) and the mean ± standard deviation (SD) of the differences. SD of MD was calculated using the following formula: SD = sqrt ((SD “baseline”) 2 + (SD “endpoint”) 2 − (2R × SD “baseline” × SD “endpoint”)), where R is the correlation coefficient. We assumed an R of 0.40 to impute the missing SD of the mean within-group change according to Follman et al. [29]. If a 95% confidence interval (95% CI) was available for the difference in means, the same standard deviation was converted as: SD = sqrt (N) × (upper limit − lower limit)/(2u) (equal to 3.96). If the sample size was small (<30 in each group), the u-value could be obtained from tables of the t distribution with degrees of freedom equal to the sample size minus 1. This calculation is appropriate for data that are at least approximately normally distributed [30]. The random effects model was used to calculate the weighted mean difference (WMD) and 95% CI, and *p* < 0.05 was considered statistically significant. The results were assessed by comparing the mean ± SD of the change in HFs of the active group with the control group [31]. Cochrane Q and I^2^ statistic were used to assess the heterogeneity. The I^2^ test assessed whether the variance across studies was correct and not a result of a sampling error. The percentage of total variation indicated the degree of heterogeneity; I^2^ values of ≤25% were considered low, >25% as moderate, and ≥75% as high heterogeneity [32]. STATISTICA Medical Software 11.0 StatSoft Poland, Krakow, Poland has been used for all statistical analyses.

## 3. Results

**Figure 1:**
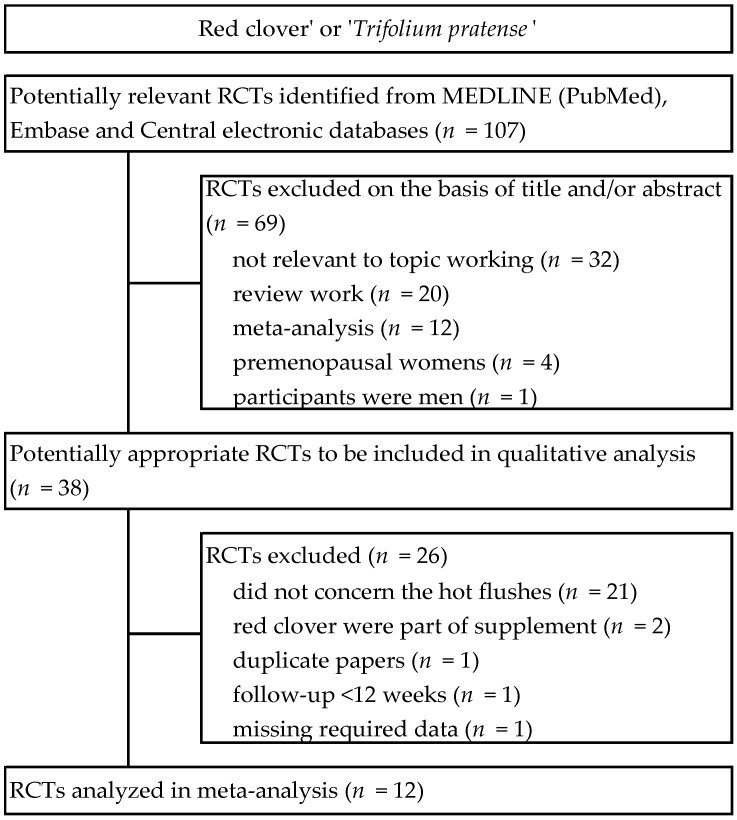
Flowchart of the selection procedure for studies included in the current review regarding red clover in menopausal symptoms. Abbreviations: RCTs, randomized controlled trials.

As a result of the search of electronic databases, 107 RCTs were identified. Sixty nine studies were excluded on the basis of title and/or abstracts. In the second phase, thirty eight potentially significant randomized controlled trials were identified and submitted for full-text assessment. Of these, twenty six studies were excluded due to failure to meet inclusion criteria. As a result, twelve RCTs that described the administration of RCIE to women for the management of HFs were included in the meta-analysis [33,34,35,36,37,38,39,40,41,42,43,44]. A detailed review of the selection procedure is shown in Figure 1.

### 3.1. Characteristics of Included Trials

The characteristic of the selected twelve randomized, placebo-controlled clinical studies are reported in Table 1. All trials used a parallel group design, with the exception of three studies that used a crossover design [34,39,41]. The trials’ duration ranged from 12 weeks to 2 years. Clinical studies were conducted in Australia (3), Peru, the Netherlands, the United States, the United Kingdom, Ecuador, Brazil, Austria, Iran, and Denmark. Overall, 1179 women experiencing menopause participated in the studies, and sample size ranged from 37 to 252 (1043 participants were included in the final analysis). Eight trials included postmenopausal women exclusively, three studies included women in both the peri- and postmenopausal period [37,38,40], and perimenopausal women were included in one study [44]. The average red clover isoflavone dose was 65.1 mg/d of aglycone equivalent (range, 37.1–160 mg/d). Two studies included two therapeutic arms with different doses of isoflavones [33,37]. The composition of the isoflavones and their doses varied among studies, which is shown in Table 1. Eight studies measured the daily frequency of HFs; the baseline of hot flushes was over three per day. Ten studies included the presence and/or severity of various somatic and psychological symptoms on scales to assess menopausal symptoms.

### 3.2. Assessment of the Methodological Quality of Trials

**Figure 2:**
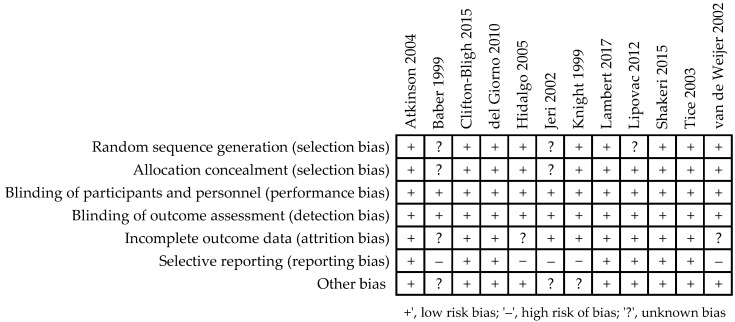
Risk of bias summary for each study as assessed by the authors [33,34,35,36,37,38,39,40,41,42,43,44].

**Figure 3:**
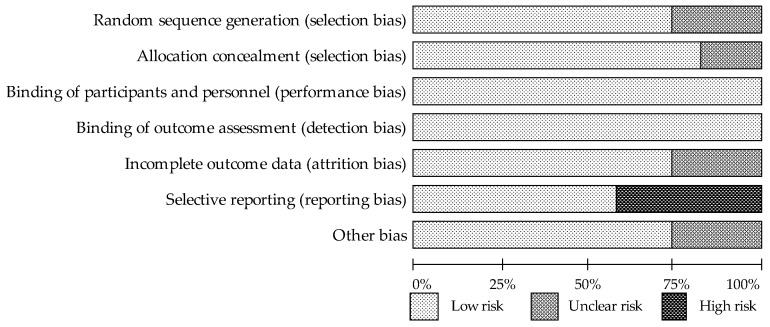
The assessment of risk of bias for each item; data are shown as a percentage for trials.

Details of the risk of bias assessment are shown in Figure 2 and Figure 3. Several trials were characterized as “unclear risk”, relating to the lack of sufficient information in the categories random sequence generation (selection bias) [34,35,41] (25%) and allocation concealment (selection bias) [34,35] (17%). The categories that presented a low risk of bias in all evaluated trials were the blinding of participants and personnel (performance bias) and the blinding of outcome assessment (detection bias). In the category of incomplete outcome data (attrition bias), “unclear bias” was demonstrated in 25% of studies [34,36,39]; it was not clear whether dropouts were likely to influence results. With respect to the selective reporting category, five studies [33,34,35,36,39] (42%) presented a “high risk of bias” associated with the lack of reports of adverse effects. “Unclear risk” in the other bias category was associated with an insufficient description of the study funding.

### 3.3. Systematic Review and Meta-Analysis

We present the results of a comprehensive systematic review with a meta-analysis regarding the impact assessment of RCIE on the incidence of hot flushes and on the presence and/or severity of various somatic and psychological symptoms in the evaluation of the menopausal symptom questionnaires and scales in perimenopausal and postmenopausal women.

#### 3.3.1. Daily Hot Flushes Frequency

In most studies, a dose of 40–80 mg/d RCIE was used, except for the study of Lambert et al. [44], in which 37.1 mg/d was administered. In two trials, parallel comparisons with other doses were also performed: 57 mg/d [33] and 180 mg/d [37].

Of the eight RCTs with ten comparisons assessing frequency of HFs, six [33,35,36,37,41,44] showed a reduction in HFs, including four significant decreases [35,36,41,44] in the isoflavone group compared to the placebo group; while in four comparisons, no differences were observed between the groups [33,34,37,38].

**Figure 4:**
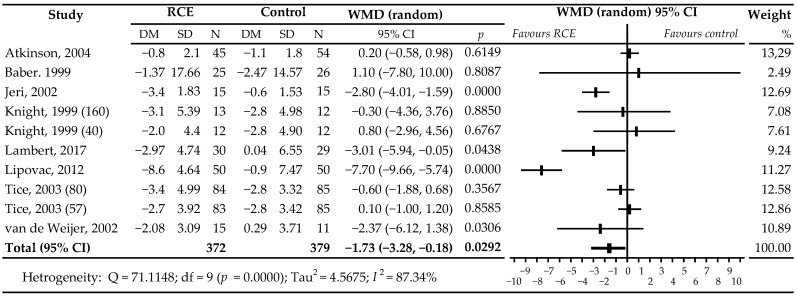
Effects of isoflavones with red clover (*Trifolium pratense*) vs. placebo on the daily frequency of hot flushes in peri- and post-menopausal women. Number in brackets following author’s name refers to dose of isoflavones in the study with more than one active group [33,34,35,36,37,38,41,44]. Abbreviations: RCIE, red clover isoflavone extract; WMD, weighted mean difference.

A meta-analysis of all comparisons showed a statistically significant reduction in the daily incidence of HFs in women receiving active treatment compared to those receiving placebo treatment: WMD −1.73 HFs/d, 95% CI −3.28 to −0.18; *p* = 0.0292 (Figure 4). Additionally, a subgroup analysis was conducted to explain the possible influence of covariates on the observed high heterogeneity of included trials (I^2^ = 87.34%) based on five prognostic factors: menopausal status, observation time, frequency of daily HFs, the total dose of isoflavones in terms of aglycone equivalents, and the differences in the types of isoflavone. Results of the sub-analysis are shown in Table 2. Differences in means were larger in comparisons that used RCIE at a dose of ≥80 mg/day (WMD −2.80 HFs/d; *p* = 0.1210), as well as when the formulations contained a higher proportion of biochanin A (−1.79 HFs/d; *p* = 0.0520), in postmenopausal women (WMD −2.68 HFs/d; *p* = 0.0105), with ≥5 HFs per day (WMD −2.56; *p* = 0.0096), and with an observation period of 12 weeks (WMD −1.95 HFs/d; *p* = 0.0206).

#### 3.3.2. Rating of Menopausal Complaints Using Instruments to Measure Intensity of Symptoms

**Figure 5:**
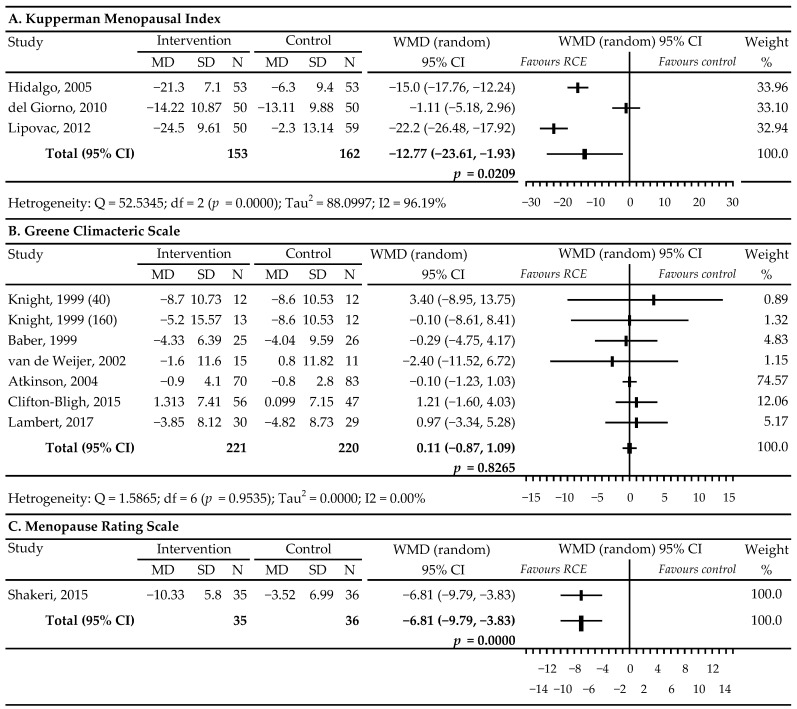
Effects of red clover (*Trifolium pratense*) isoflavones vs. placebo on rating menopausal symptoms using the following questionnaires, based on the respondents’ replies concerning the intensity of complaints. Number in brackets following author’s name refers to the dose of isoflavones in the study with more than one active group [33,34,36,38,39,40,41,42,43,44]. The letter A marks the first part of the figure containing the Kupperman Menopausal Index analysis. The letter B marks the second part of the figure containing the Greene Climacteric Scale analysis. The letter C marks the third part of the figure containing the Menopause Rating Scale analysis. Abbreviations: WMD, weighted mean difference.

In three studies [39,40,41], the Kupperman Menopausal Index (KMI) scale was used to assess the severity of climacteric symptoms. The results of the meta-analysis indicated that compared to placebo, RCIE was effective in relieving menopausal symptoms: WMD −12.77, 95% CI −23.61 to −1.93; *p* = 0.0209; I^2^ = 96.19% (Figure 5A). Of the six RCTs with seven comparisons that reported Greene Climacteric Scale (GCS) data, four (40 mg/d) [33,34,37,38] showed a marginal, insignificant decrease in GCS scores, in contrary to three studies (160 mg/d) [33,42,44] that reported a slight, insignificant intensification of menopausal symptoms. Meta-analysis did not show the beneficial effect of RCIE on menopausal symptoms and complaints in the GCS scale used in these studies, compared with the placebo: WMD 0.11, 95% CI −0.87 to 1.09; *p* = 0.8265; I^2^ = 0.00% (Figure 5B).

According to the results obtained from a single RCT [43], women receiving RCIE observed a significant reduction in their Menopause Rating Scale (MRS) score compared to placebo: WMD −6.81, 95% CI −9.79 to −3.83; *p* = 0.0000 (Figure 5C).

## 4. Discussion

Our meta-analysis of all comparisons showed a statistically significant reduction in the daily incidence of HFs in women receiving active treatment compared to those receiving placebo, WMD −1.73, 95% CI −3.28 to 0.18; *p* = 0.0292. These results are consistent with those presented in previous meta-analyses [45,46,47] that also found some beneficial effects of RCIE, although not always statistically significant. Myers and Vigar [45], based on the analysis of 5 studies [33,36,37,39,41] including 438 women, showed a statistically significant reduction in the number of HFs after a daily intake of ≥80 mg RCIE compared to placebo: WMD −3.46, 95% CI −4.37 to −2.56; *p* < 0.00001. Coon et al. [46] reported a significant decrease in the daily episodes of flushing (WMD −1.63, 95% CI −2.97 to −0.28; *p* < 0.02) in an analysis that included 5 trials [33,34,35,36,37] with 7 comparisons and 385 participants; the doses of RCI were 40, 57, 80–82, or 160 mg/d. In turn, a meta-analysis by Ghazanfarpour et al. [47] based on 6 studies [33,34,35,36,37,41] showed a decrease in HFs/day frequency, close to statistical significance, post-administration of RClE in 40 or 80 mg: WMD −1.99, 95% CI −4.12 to 0.19; *p* = 0.067. Furthermore, three meta-analyses [48,49,50] showed positive effects of RClE, but they were statistically non-significant on the frequency of HFs. Lethaby et al. [48], using five studies [33,34,35,36,37], showed a slight decrease, WMD −0.93, 95% CI −1.95 to 0.10; *p* = 0.21 (40 mg/d, 80 mg/d doses). In turn, Nelson et al. [49] reported a reduction in HFs (WMD = −0.44, 95% CI −1.47 to 0.58) based on 9 comparisons (6 studies [33,34,35,36,37,38]; doses of 40 mg/d, 57 mg/d, 80–82 mg/d, 160 mg/d). While Franco et al. [50] noted a decrease in the number of daily hot flushes for red clover isoflavones compared with placebo: WMD = −1.84, 95% −3.87 to 0.19; *p* = 0.20 (7 studies [33,34,35,36,37,38,41]; doses: 40–160 mg).

The discussion omitted the results of systematic reviews with a meta-analysis [51,52] with the adopted research methodology based on the analysis of only the final values of HFs at the end of treatment.

Our meta-analysis also showed that RCIE significantly lowered the KMI points—WMD −12.77 (*p* = 0.0209) and the MRS points—WMD −6.81 (*p* = 0.0000). The last result was only based on one study [43], which makes it impossible to draw a final conclusion. The earlier meta-analysis of Myers and Vigor [45] assessing the effect of RCIE on menopausal symptoms on the KMI scale, based on two trials [39,41], also demonstrated a marked significant reduction of WMD −21.8 points (*p* < 0.00001) in women receiving red clover. It is important to note that the lack of a significant reduction on the GCS (WMD 0.11 points; *p* = 0.8265) found in our analysis may undermine the usefulness of RCIE in relieving menopausal symptoms other than HFs in perimenopausal and postmenopausal women.

A number of possible limitations should be taken into account when interpreting the results of the present meta-analysis. The fact that most of these assessments were based on a relatively limited number of available trials as well as the small number of participants in some studies may result in insufficient statistical power and limit the drawdown of definitive conclusions. Also, inter-individual differences in the metabolism and bioavailability of isoflavones may cause variability in the response to their use. The placebo effect should also be taken into account: clear placebo responses in menopausal women in drug trials affecting vasomotor symptoms are well documented [53]. Furthermore, the actual dose of aglycone isoflavones administered or absorbed was difficult to determine. Other limitations are related to the fact that the analyzed works may not represent all research related to this topic, especially articles published in languages other than English. Additionally, if the error of published research is strong, it is possible to have overestimated or underestimated the effect of red clover on menopausal symptoms.

## 5. Conclusions

This meta-analysis of randomized controlled trials assessing the effect of a specific standardized extract of red clover isoflavones on menopausal symptoms showed a statistically moderate relationship with the reduction in the daily frequency of hot flushes. However, further well-designed studies are required to confirm the present findings and to finally determine the effects of red clover on the relief of flushing episodes, to provide more comprehensive information about well-defined preparations, and the optimal dose and duration of taking red clover aglycones to achieve their highest effectiveness.

## Figures and Tables

**Table 1:** Randomized controlled trials of Trifolium pratense for alleviating menopause symptoms: studies’ characteristics.

First Author Pub. Data (Ref.) Country	Design Follow-up Period	Sample Size: Randomized/Analyzed	Participants Age, y (Range) Trial Inclusion Criteria	Intervention: Isoflavone Daily Dose	Baseline Hot Flush Frequency/d	Baseline Menopausal Score
Knight 1999 [33] Australia	Placebo controlled 3-arm parallel trial 1 wk placebo run-in/12 wk follow-up	37/37	54.6 ± 3.6 (40–65) Healthy postmenopausal women, bilateral oophorectomy or amenorrhea ≥ 6 mo, FSH > 40 mIU/mL, HF > 3/d	RCG “a”; 160 mg^a^ RCG “b”: 40 mg^b^ PG: placebo	RCG “a”: 9.0 ± 5.2 RCG “b”: 6.9 ± 2.1 PG: 8.6 ± 4.6	GCS RCG “a”: 19.9 ± 4.4 RCG “b”: 19.9 ± 10.6 PG: 18.5 ± 11.4
Baber 1999 [34] Australia	Placebo controlled crossover trial 90 d active phase/ 7 d washout	51/51	54.0 ± 4.1 (45–65) Healthy postmenopausal women, age of menopause 50.0 ± 3.6 y, FSH > 30 mIU/mL, HF > 3/d	RCG: 40 mg^b^ PG: placebo	RCG: 6.2 ± 2.7 PG: 6.4 ± 2.6	GCS RCG: 10.9 ± 6.5 PG: 12.3 ± 9.0
Jeri 2002 [35] Peru	Placebo controlled parallel trial 16 wk follow-up	30/30	51.0 ± 3.5 (<60) Healthy postmenopausal women, amenorrhea ≥ 12 mo, FSH > 30 mIU/mL, HF ≥ 5/d	RCG: 40 mg^b^ PG: placebo	RCG: 7.0 ± 1.9 PG: 5.7 ± 1.6	- -
van de Weijer 2002 [36] Netherlands	Placebo controlled parallel trial 4 wk placebo run-in/ 12 wk follow-up	30/26	53.4 ± 6.3 (49–65) Healthy postmenopausal women, amenorrhea ≥ 12 mo, BMI 26.1 ± 4.2, HF ≥ 5/d	RCG: 80 mg^c^ PG: placebo	RCG: 5.43 ± 2.6 PG: 5.6 ± 5.0	GCS RCG: 12.5 ± 11.2 PG: 13.8 ± 9.5
Tice 2003 [37] United States	Placebo controlled 3-arm parallel trial 2 wk placebo run-in/12 wk follow-up	252/252	52.3 ± 3.1 (45–60) Healthy peri- and post-menopausal women, 3.3 ± 4.5 ysm, FSH > 30 mIU/mL, BMI 26.1 ± 4.9, HF ≥ 35/wk	RCG “a”: 80 mg^c^ RCG “b”: 57 mg^d^ PG: placebo	RCG “a”: 8.5 ± 5.8 RCG “b”: 8.1 ± 3.0 PG: 7.8 ± 2.4	- - -
Atkinson 2004 [38] United Kingdom	Placebo controlled parallel trial 12 mo follow-up	205/99	52.2 ± 4.8 (49–65) Healthy peri- and post-menopausal women, FSH > 30 mIU/mL, BMI 25.3 ± 3.7, HF > 3/d	RCG: 40 mg^e^ PG: placebo	RCG: 2.1 ± 2.7 PG: 2.5 ± 3.0	GCS RCG: 4.3 ± 4.3 PG: 4.3 ± 4.3
Hidalgo 2005 [39] Ecuador	Placebo controlled crossover trial 90 d active phase/ 7 d washout	60/53	51.3 ± 3.5 (>40) Healthy postmenopausal women, amenorrhea ≥ 12 mo, FSH > 35 mIU/ml, BMI 26.6 ± 3.9,KMI score ≥ 15	RCG: 80 mg^c^ PG: placebo	- -	KMI RCG: 27.2 ± 7.7 PG: 27.2 ± 7.7
del Giorno 2010 [40] Brazil	Placebo controlled parallel trial 12 mo follow-up	120/100	55.5 ± 4.9 (45–65) Healthy peri- and post-menopausal women, amenorrhea ≥ 12 mo, FSH > 30 mIU/mL, BMI 28.8 ± 5.4	RCG: 40 mg PG: placebo	- -	KMI RCG: 25.3 ± 10.2 PG: 25.1 ± 9.0
Lipovac 2012 [41] Austria	Placebo controlled crossover trial 90 d active phase/ 7 d washout	113/109	54.1 ± 7.0 (>40) Healthy postmenopausal women, amenorrhea ≥ 12 mo, FSH > 35 mIU/mL, BMI 24.7 ± 3.9, HF > 5/d, KMI score ≥ 15/wk	RCG: 80 mg^c^ CG: placebo	RCG: 11.7 ± 4.8 PG: 11.0 ± 5.1	KMIRCG: 32.5 ± 10.0 PG: 34.3 ± 10.4
Clifton-Bligh 2015 [42] Australia	Placebo controlled parallel trial 1 mo placebo run-in/ 2 y follow-up	147/103	54.4 ± 3.9 Healthy postmenopausal women, amenorrhea ≥ 12 mo, FSH > 30 mIU/mL, BMI 24.8 ± 4.3	RCG: 57 mg^d^ PG: placebo	- -	GCS RCG: 8.9 ± 7.3 PG: 11.0 ± 8.0
Shakeri 2015 [43] Iran	Placebo controlled parallel trial 12 wk follow-up	72/71	54.8 ± 2.8 (50–59) Healthy postmenopausal women, 1.85 ± 0.9 ysm, BMD 21.1 ± 1.9	RCG: 80 mg^c^ PG: placebo	- -	MRS RCG: 20.4 ± 6.3 PG: 20.8 ± 6.2
Lambert 2017 [44] Denmark	Placebo controlled parallel trial 12 wk follow-up	62/59	52.5 ± 3.5 (40–65) Healthy perimenopausal women, FSH ≥ 35 mIU/mL, BMI 25.7 ± 4.3, HF > 5/d	RCG: 37.1 mg^f^ CG: placebo	RCG: 9.5 ± 6.4 PG: 8.6 ± 6.9	GCS RCG: 18.6 ± 12.3 PG: 20.8 ± 2.3

Data are presented as mean ± standard deviation. Abbreviations: -, data not available; BIO, biochanin A; BMI, body mass index (kg/m^2^); DAI, daidzein; FOR, formononetin; FSH, follicle-stimulating hormone; GCS, Greene Climacteric Scale; GEN, genistein; GLY, glycitein; HF, hot flushes; KMI, Kupperman Menopausal Index; MRS, Menopause Rating Scale; PG, placebo group; pub. data, publication data; RCG, red clover group; ref., reference; VAS, vasomotor symptoms; ysm, years since menopause; mo, months; wk, week; d, day. Composition of isoflavones (aglycone, mg): ^a^ BIO (98.0), FOR (32.0), GEN (16.0), DAI (14.0); ^b^ BIO (24.5), FOR (8.0), GEN (4.0), DAI (3.5); ^c^ BIO (49.0), FOR (16.0), GEN (8.0), DAI (7.0); ^d^ FOR (44.6), BIO (5.8), DAI (1.8), GEN (0.8), GLY (0.8); ^e^ BIO (26.0), FOR (16.0), GEN (1.0), DAI (0.5); ^f^ FOR (19.0), BIO (9.0), GEN (4.2), DAI (1.6).

**Table 2:** Assessment of the effect of red clover isoflavones on the frequency of hot flushes in subgroup analysis.

Variables	*n*	Simple Size	WMD (95% CI)	*p*	*I*^2^ (%)
**Overall effects**	10	751	−1.73 (−3.28, −0.18)	0.0292	87.34
**Menopausal status**					
Postmenopausal	7	315	−2.68 (−4.72, −0.63)	0.0105	71.44
Peri- and post-menopausal	3	436	0.01 (−0.55, 0.58)	0.9594	0.00
**Follow-up period**					
12 weeks	9	652	−1.95 (−3.61, −0.30)	0.0206	81.33
12 months	1	99	0.20 (−0.58, 0.98)	0.6149	(-)
**Frequency of hot flushes**					
≥5/day	6	552	−2.56 (−4.49, −0.62)	0.0096	87.67
≥3/day	4	199	0.21 (−0.53, 0.96)	0.5761	0.00
**Isoflavones dose**					
<80 mg/day	6	431	−0.88 (−2.34, 0.58)	0.2370	76.83
≥80 mg/day	4	320	−2.80 (−6.35, 0.74)	0.1210	86.61
**Dominant of isoflavones**					
Biochanin A	8	524	−1.79 (−3.60, 0.02)	0.0520	85.78
Formononetin	2	227	−1.14 (−4.13, 1.84)	0.4519	73.64

*n*, number of comparisons; -, not calculated.

## Data Availability

Not applicable.
